# Lower Plasma Total Testosterone Levels Were Associated With Steeper Decline in Brain Glucose Metabolism in Non-demented Older Men

**DOI:** 10.3389/fnagi.2021.592845

**Published:** 2021-04-15

**Authors:** Xiwu Wang, Zhaoting Lv, Qian Wu, Huitao Liu, Yanrou Gu, Teng Ye

**Affiliations:** ^1^Department of Psychiatry, Wenzhou Seventh People’s Hospital, Wenzhou, China; ^2^Department of Psychiatry, Tongde Hospital of Zhejiang Province, Hangzhou, China; ^3^School of Mental Health, Wenzhou Medical University, Wenzhou, China; ^4^Department of Orthopedics, Taizhou Hospital of Zhejiang Province, Taizhou, China; ^5^Department of Ultrasound, The First Affiliated Hospital of Wenzhou Medical University, Wenzhou, China

**Keywords:** testosterone, Alzheimer’s disease, brain glucose metabolism, FDG, longitudinal study

## Abstract

**Objective:**

There is growing evidence that testosterone may be implicated in the pathogenesis of Alzheimer’s disease (AD). We aimed to examine the relationship between plasma total testosterone levels and change in brain glucose metabolism over time among non-demented older people.

**Methods:**

The association of plasma total testosterone levels with change in brain glucose metabolism among non-demented older people was investigated cross-sectionally and longitudinally. Given a significant difference in levels of plasma total testosterone between gender, we performed our analysis in a sex-stratified way. At baseline, 228 non-demented older people were included: 152 males and 76 females.

**Results:**

In the cross-sectional analysis, no significant relationship between plasma total testosterone levels and brain glucose metabolism was found in males or females. In the longitudinal analysis, we found a significant association of plasma total testosterone levels with change in brain glucose metabolism over time in males, but not in females. More specifically, in males, higher levels of total testosterone in plasma at baseline were associated with slower decline in brain glucose metabolism.

**Conclusion:**

We found that higher levels of total testosterone in plasma at baseline were associated with slower decline in brain glucose metabolism in males without dementia, indicating that testosterone may have beneficial effects on brain function.

## Introduction

There is growing evidence that testosterone may be implicated in the pathogenesis of Alzheimer’s disease (AD) (Holland et al., 2011). For example, males with AD and other dementias showed lower levels of total testosterone in serum compared to controls (Bowen et al., 2000; Hogervorst et al., 2001, 2003; Moffat et al., 2004; Paoletti et al., 2004). A number of cross-sectional studies have also suggested that testosterone levels are positively associated with memory performance, such as visual, working, and verbal memory (Barrett-Connor et al., 1999; Muller et al., 2005; Thilers et al., 2006; Van Strien et al., 2009). Additionally, the Baltimore Longitudinal Study of Aging (BLSA) with an average follow-up time of 19.1 years suggests that higher levels of free testosterone in serum at baseline are associated with lower risk of developing AD dementia (Moffat et al., 2004). Similarly, animal studies indicate a beneficial role of testosterone against tau phosphorylation (Papasozomenos and Shanavas, 2002) and β-amyloid (Aβ) production (Gouras et al., 2000). Further, Sundermann et al. (2020) found that lower testosterone levels in plasma were associated with higher CSF p-tau levels among APOE4 carriers. A recent meta-analysis of randomized clinical trials investigating the effects of testosterone supplementation on cognition suggested that testosterone supplementation may be a potentially preventative measure again cognitive impairment among cognitively normal older people (Tan et al., 2019). Taken together, these studies suggest that testosterone plays an important role in the pathogenesis of AD and may be neuroprotective.

Despite numerous studies supporting the notion that testosterone may affect cognitive performance and the risk of developing AD, the evidence on the relationship between testosterone and brain physiology is limited. In a cross-sectional study, Moffat and Resnick (2007) found that high circulating testosterone levels were positively associated with cerebral blood flow and brain glucose metabolism, which can be used to predict cognitive and functional decline in patients with cognitive impairment and AD (Landau et al., 2011). However, cross-sectional examination of the relationship between testosterone and brain glucose metabolism prohibits us from clarifying the temporal association of testosterone with brain glucose metabolism. Additionally, no previous studies have examined the relationship between plasma total testosterone levels and change in brain glucose metabolism over time among non-demented older people. If this relationship exists, it may provide novel insights into the effect of testosterone on brain function.

In the current study, we aimed to examine the association of plasma total testosterone levels at baseline with change in brain glucose metabolism over time among non-demented older people. Further, given the difference in levels of plasma total testosterone between gender, sex-stratified models were performed in males and females separately.

## Materials and Methods

### Alzheimer’s Disease Neuroimaging Initiative (ADNI)

Data used in the present study were downloaded from the Alzheimer’s Disease Neuroimaging Initiative (ADNI), which was launched in 2003^1^. The primary aim of the ADNI study has been to examine whether a variety of predictors, such as cognitive assessments, neuroimaging markers, and fluid biomarkers, can be integrated to assess the progression of mild cognitive impairment (MCI) and early AD. At every ADNI center, institutional review board approved the study, and each participant provided informed consent. In the present study, we selected 228 non-demented older people, including 26 individuals with normal cognition and 202 individuals with MCI. Participants with normal cognition had a Clinical Dementia Rating (CDR) (Morris, 1993) score of 0 and a Mini-Mental State Examination (MMSE) (Folstein et al., 1975) score of 24 or higher. Individuals with MCI had an MMSE score of 24 or higher, a CDR score of 0.5, objective memory impairment as examined by the Wechsler Memory Scale Logical Memory II, and an absence of dementia.

**FIGURE 1:**
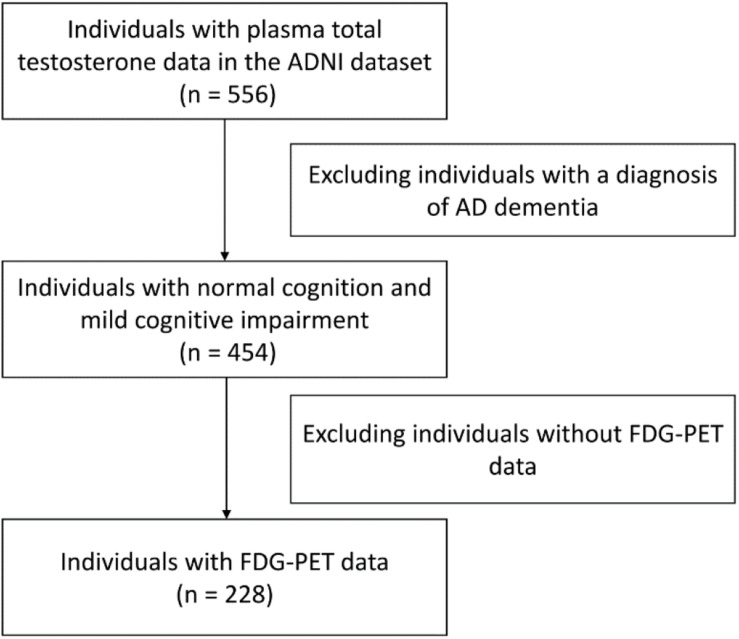
A flowchart of the data selection at baseline.

Demographical and clinical variables were obtained from the ADNI database. A flowchart of the data selection is presented (Figure 1).

### Measurement of Brain Glucose Metabolism

Brain glucose metabolism was examined by FDG-PET. A standard protocol was used to process images^2^. ADNI researchers established a “MetaROI” of brain regions based on the finding that these regions show metabolic alterations in MCI or AD subjects which are closely associated with cognitive performance (Jagust et al., 2010; Landau et al., 2011). This MetaROI was made up of five regions, including Left Angular Gyrus, Right Angular Gyrus, Bilateral Posterior Cingular, Left Inferior Temporal Gyrus, and Right Inferior Temporal Gyrus. Standardized uptake value ratios (SUVRs) were used as dependent variable in our models. SUVRs were calculated by the formula: SUVRs = the average of FDG uptake of five MetaROI/the reference region (pons and cerebellum).

### Measurement of Plasma Total Testosterone Levels

Plasma total testosterone levels were measured by a multiplex-based immunoassay panel developed by Rules-Based Medicine (MyriadRBM), details of which can be found at the website^3^. Values are given in ng/ml. Plasma total testosterone data were log10-transformed in order to normalize the distribution before entering our analysis.

### Statistical Analysis

The *t*-test was utilized to determine the differences in continuous variables (age, education, MMSE, and plasma total testosterone levels). The chi-square test was used to compare the distribution of categorical variable (APOE4 genotype) between males and females. To examine the association of plasma total testosterone levels with change in brain glucose metabolism over time, linear mixed models were performed with FDG SUVRs as dependent variable. Given that males showed significantly higher levels of plasma total testosterone than females (mean: 0.43 vs −0.36), sex-stratified analyses were conducted. In males, levels of plasma total testosterone were categorized into two groups based on the median (0.48). In females, levels of plasma total testosterone were categorized into two groups based on the median (−0.26). Thus, plasma total testosterone was regarded as categorical variable in our models. All models included main effects of plasma total testosterone (in males: ≤0.48 vs >0.48; in females: ≤−0.26 vs >0.26), age, APOE4 genotype, education, and their interactions with time. In addition, models also included a random intercept for each subject. In our linear mixed models, a row of the long-format data frame that has a missing value was removed from the analysis. When missing values occurred for FDG SUVRs (the dependent variable) during follow-up visits, a participant was included in our linear mixed model if the participant had one or more non-missing time point.

All statistical work was conducted using R software (version 4.0.0) (R Core Team, 2013).

## Results

### Demographics and Clinical Variables by Gender

Table 1 shows the demographical and clinical information of 228 non-demented older people: 152 males and 76 females. There were no significant differences in age, education, APOE4 genotype, MMSE, or FDG SUVRs between males and females (all *p* > 0.05). However, males showed significantly higher levels of plasma total testosterone than females (*p* < 0.001). The number of participants at follow-up visits is also displayed in Table 1.

**TABLE 1:** Demographics and clinical variables by gender.

Variables	Males (*n* = 152)	Females (*n* = 76)	*P*-values
Age, years	75.3 ± 7.03	74.3 ± 7.04	0.33
Education, years	15.8 ± 2.92	15.7 ± 2.86	0.81
APOE4 carriers, *n* (%)	75 (49.3)	35 (46.1)	0.64
MMSE	27.3 ± 1.7	27.4 ± 1.81	0.62
FDG SUVRs	1.21 ± 0.13	1.21 ± 0.14	0.79
Plasma total testosterone^b^ (log10-transformed), ng/ml	0.43 ± 0.19	−0.36 ± 0.37	<0.001
**The number of subjects at each visit point^a^**			
Baseline	152	76	
0.5	142	70	
1	128	68	
1.5	101	51	
2	106	58	
3	89	43	
4	47	32	
5	36	15	
6	27	20	
7	21	11	
8	2	4	
10	2	4	
11	3	5	
12	1	1	

*MMSE, Mini-Mental State Examination; FDG SUVRs, fluorodeoxyglucose standardized uptake value ratios. ^*a*^Participants who had FDG-PET measured were included. ^*b*^The non-transformed mean values for males and females were 2.94 ± 1 (mean ± SD) ng/ml and 0.57 ± 0.36 ng/ml, respectively.*

### Cross-Sectional Association of Plasma Total Testosterone Levels With FDG SUVRs in Males and Females

**FIGURE 2:**
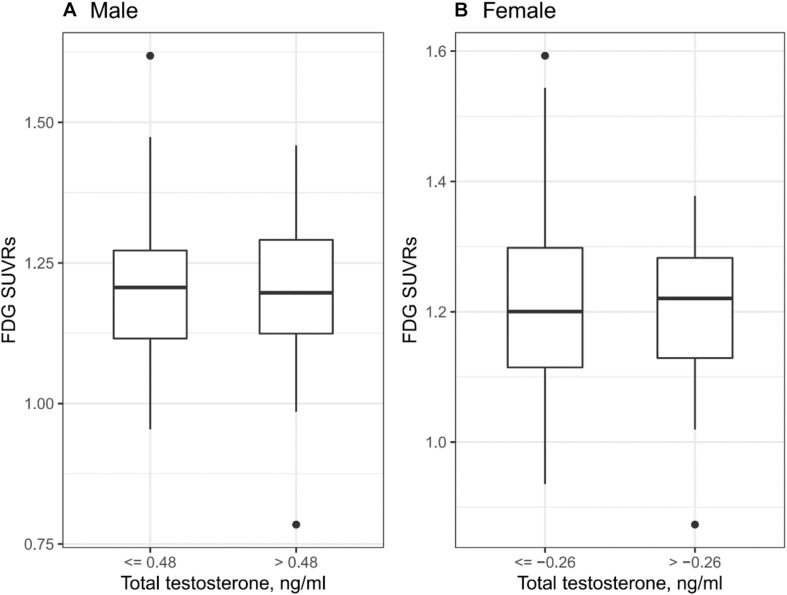
Cross-sectional relationship between plasma testosterone and FDG SUVRs. **(A)** In males, there was no statistically significant relationship between plasma total testosterone levels and FDG SUVRs (*p* > 0.05). **(B)** In females, there was no statistically significant relationship between plasma total testosterone levels and FDG SUVRs (*p* > 0.05).

*T*-tests were conducted to examine the relationship between plasma total testosterone levels and FDG SUVRs. Given that males showed significantly higher levels of plasma total testosterone than females (mean: 0.43 vs −0.36, Table 1), sex-stratified analyses were conducted. In males, levels of plasma total testosterone were categorized into two groups based on the median (0.48). In females, levels of plasma total testosterone were categorized into two groups based on the median (−0.26). However, there was no statistically significant relationship between plasma total testosterone levels and FDG SUVRs in males or females (all *p* > 0.05, Figure 2).

### Longitudinal Association of Plasma Total Testosterone Levels With FDG SUVRs in Males and Females

**FIGURE 3:**
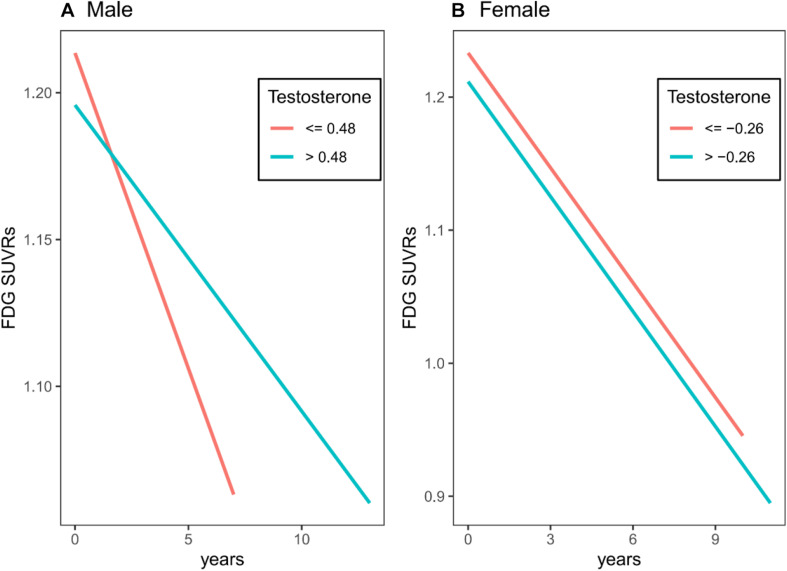
Longitudinal association of plasma total testosterone levels with FDG SUVRs in males and females. **(A)** In males, higher plasma testosterone levels were significantly associated with slower decline in FDG SUVRs. **(B)** In females, we did not find a relationship between plasma total testosterone levels and change in FDG SUVRs over time.

To examine the longitudinal association of plasma total testosterone levels with FDG SUVRs, linear mixed models were conducted. In males, higher plasma testosterone levels were significantly associated with slower decline in FDG SUVRs (estimate: 0.012, *p* < 0.001, Table 2 and Figure 3A). However, in females, we did not find a relationship between plasma total testosterone levels and change in FDG SUVRs over time (estimate: −0.0013, *p* = 0.7269, Table 3 and Figure 3B).

**TABLE 2:** Results of linear mixed models in males.

Predictors	Estimate	SE	*P*-value
Higher testosterone × time	0.012	0.003	<0.001
Age × time	0.0006	0.0002	0.0001
Education × time	−0.0006	0.0003	0.0535
APOE4 carriers × time	−0.0034	0.0024	0.1547

*This model included main effects of plasma total testosterone, age, APOE4 genotype, and education, while their estimates were not shown.*

**TABLE 3:** Results of linear mixed models in females.

Predictors	Estimate	SE	*P*-value
Higher testosterone × time	−0.0013	0.0038	0.7269
Age × time	−0.0002	0.0003	0.4195
Education × time	−0.0028	0.0007	0.0001
APOE4 carriers × time	−0.0274	0.0046	<0.0001

*This model included main effects of plasma total testosterone, age, APOE4 genotype, and education, while their estimates were not shown.*

## Discussion

In this study, we examined the relationship between plasma total testosterone levels and brain glucose metabolism both cross-sectionally and longitudinally among non-demented older people. Given a significant difference in levels of plasma total testosterone between gender, the analysis was performed in a sex-stratified way. In the cross-sectional analysis, we did not find a significant relationship between plasma total testosterone levels and brain glucose metabolism in males or females. In the longitudinal analysis, we found a significant relationship between plasma total testosterone levels and change in brain glucose metabolism over time in males, but not in females. More specifically, in males, higher levels of total testosterone in plasma at baseline were associated with slower decline in brain glucose metabolism, indicating that testosterone may have beneficial effects on brain function.

The key finding that higher plasma total testosterone levels at baseline were associated with slower decline in brain glucose metabolism among non-demented older men is in line with other previously published findings showing that testosterone may have protective effects on brain function. For instance, compared to healthy controls, patients with dementia showed significantly lower testosterone levels in serum (Bowen et al., 2000). In addition, several longitudinal studies suggested that reduced testosterone is an independent risk factor for developing AD (Moffat et al., 2004; Chu et al., 2010), and that higher testosterone may thus protect against cognitive impairment and AD dementia. Although emerging evidence suggests a relationship between higher testosterone and better cognitive performance and lower risk of developing AD dementia, the evidence regarding the effect of testosterone on brain glucose metabolism is limited. Our study, for the first time, provides evidence that higher testosterone may slow down the process of brain glucose hypometabolism among non-demented older men.

It is also biologically plausible that testosterone could have some beneficial effects on brain function. First, it is possible that testosterone may reduce levels of amyloid plaques and tangles in the brain, thus contributing to higher brain glucose metabolism. For example, animal studies indicate a beneficial role of testosterone against tau phosphorylation (Papasozomenos and Shanavas, 2002) and Aβ production (Gouras et al., 2000). Second, testosterone has been reported to be beneficial to human primary neurons, and this beneficial effect is not dependent on estrogen action (Hammond et al., 2001). Third, in hippocampal CA1 neurons, androgen administration can protect against NMDA excitotoxicity and may accelerate the process of recovery after injury through enhancing fiber outgrowth (Morse et al., 1992).

This study has several limitations. First, we cannot provide a causal evidence due to the observational nature of this study. Clinical trials are required to examine whether testosterone could slow down the speed of brain glucose hypometabolism and thus protect against AD. Second, given that the ADNI study represents a convenience sample of volunteers, selection bias should be considered. Third, in ADNI, plasma levels of total testosterone were measured on the Luminex xMAP platform by Biomarkers Consortium Plasma Proteomics Project Rules-Based Medicine multiplex, but not measured by other widely used and well-validated methods, such as isotope dilution liquid chromatography tandem mass spectrometry. Therefore, our findings should be interpreted with caution, and other studies are needed to replicate our findings in the future.

In conclusion, we found that higher levels of total testosterone in plasma at baseline were associated with slower decline in brain glucose metabolism in males without dementia, indicating that testosterone may have beneficial effects on brain function.

## Data Availability Statement

The ADNI data presented in this article are publicly accessible. Requests to access the datasets should be directed to http://adni.loni.usc.edu/.

## Ethics Statement

At every ADNI center, institutional review board approved the study (adni.loni.usc.edu). The patients/participants provided their written informed consent to participate in this study.

## Author Contributions

TY and XW designed and supervised the study. XW, ZL, QW, HL, and YG performed the research, analyzed the data, and wrote the manuscript. All authors approved the final version of manuscript.

## Conflict of Interest

The authors declare that the research was conducted in the absence of any commercial or financial relationships that could be construed as a potential conflict of interest.

## Footnotes

1 adni.loni.usc.edu

2 http://adni.loni.usc.edu/methods/pet-analysis/pre-processing/

3 https://adni.loni.usc.edu/wp-content/uploads/2010/11/BC_Plasma_Proteomics_Data_Primer.pdf
